# Putrescine Upregulates Melanogenesis Through Modulation of MITF Transcription Factor in B16F1 Mouse Melanoma Cells

**DOI:** 10.17113/ftb.62.01.24.8120

**Published:** 2024-03

**Authors:** Natchanok Talapphet, Moon-Moo Kim

**Affiliations:** Department of Applied Chemistry, Dong-Eui University, Busan 614-714, Republic of Korea

**Keywords:** B16F1 cells, melanogenesis, melanoma cells, polyamine, putrescine

## Abstract

**Research background:**

Ageing is a biochemical, metabolic and genetic physiological phenomenon. The suppression of melanin biosynthesis, evident in the greying of the hair, is a hallmark of ageing resulting from translation failure, reduced enzyme activity and cellular senescence. Putrescine, the smallest member of the polyamine family and an organic chemical, is present in living mammalian cells and plays a crucial role in regulating skin melanogenesis. Therefore, the purpose of this study is to explore the effect of putrescine on the signalling pathways of melanogenesis in melanoma cells.

**Experimental approach:**

Melanin production capacity of putrescine was analysed using a tyrosinase activity assay. To assess the cell viability of B16F1 cells exposed to putrescine, a tetrazolium dye MTT assay was performed. The effect of putrescine on melanin synthesis in the presence of H_2_O_2_ was evaluated using various *in vitro* assays in B16F1 cells. The effect of putrescine on melanin production in B16F1 cells was determined using a specific melanin production assay. Gene expression was analysed using real-time polymerase chain reaction (RT-PCR). Furthermore, the effect of putrescine on the expression of proteins related to melanin production in the cells treated with H_2_O_2_ was analysed by immunofluorescence and Western blot analysis.

**Results and conclusions:**

Putrescine increased tyrosinase activity and showed no cytotoxicity in B16F1 cells. In addition, putrescine effectively scavenged H_2_O_2_, as shown by the reduction of intracellular H_2_O_2_ amounts in 2',7'-dichlorofluorescin diacetate analysis, and promoted melanin production in living cells. The stimulation of melanogenesis by putrescine was attributed to the increased expression of *Mitf*, *Tyr*, *Trp-1* and *Trp-2* genes. Immunofluorescence assays revealed that putrescine enhanced the expression of proteins associated with melanogenesis and upregulated TYR, TRP-1 and TRP-2 *via* the microphthalmia-associated transcription factor (MITF) and increased the expression of methionine sulfoxide reductases A (MSRA) and B (MSRB) in the cells treated with H_2_O_2_, effectively promoting melanogenesis. These results suggest that putrescine can be used to stimulate melanin synthesis.

**Novelty and scientific contribution:**

This is the first study to investigate the effect of putrescine on the signalling pathways of melanogenesis in B16F1 melanoma cells. The results confirm that putrescine can promote melanogenesis through the expression of TYR, TRP-1 and TRP-2 *via* the MITF in cells treated with H_2_O_2_. Putrescine can be used exclusively as a cosmetic product to prevent premature greying of hair.

## INTRODUCTION

Melanin pigments are an essential component of the signalling system. They are derived from the amino acid l-tyrosine and are the result of intricate metabolic processes that control the synthesis of skin, eye and hair pigments (*1*). In melanocytes, the synthesis of melanin is stimulated by the interaction between the melanocyte surface receptor and alpha-melanocyte-stimulating hormone (α-MSH) (*2*). Melanocytes produce melanin, a pigment transported to keratinocytes by vesicles called melanosomes at the epidermal-dermal junction of the skin (*3*). Recent research has clearly shown that senescence supresses the cancer development, including melanoma (*4*). Melanomas arise from the malignant proliferation of melanocytes, pigment-producing cells of the skin located in the basal layer of the epidermis (*5*). Melanomagenesis, which describes the pathophysiology of melanoma, is based on sequential changes in specific genes and pathways that regulate important cell processes and influence metabolic or molecular mechanisms (*6*). The primary function of melanin is to protect DNA from UV radiation and to act as an antioxidant against reactive oxygen species (ROS), which induce cellular damage and oxidative stress (*7*). However, changes in genetic physiology and biochemical metabolism often occur during the ageing process. Ageing phenotypes are an inevitable aspect of human life. The accumulation of translational defects and cellular damage combined with reduced activity of enzymes involved in cell metabolism are the main characteristics of cellular ageing (*8*). Melanin content decreases with age, leading to dysfunctional cells and disrupted homeostasis, causing hair to turn grey. In addition, previous research has shown that oxidative stress contributes significantly to the development of melanoma (*9*).

Tyrosinase (TYR), a copper-containing metalloprotein as well as tyrosinase-related protein 1 (TRP-1) and TRP-2 play a crucial role in melanin synthesis (*10*). In addition, the microphthalmia-associated transcription factor (MITF) is essential for melanocytes and melanogenesis differentiation by regulating multiple pigmentation-related genes, including TYR, TRP-1 and TRP-2 (*11*). However, an increase in hydrogen peroxide leads to a decrease in antioxidant enzymes, including methionine sulfoxide reductase A and B (MSRA and MSRB). This disruption impairs the normal synthesis of melanogenesis and tyrosinase, resulting in depigmentation (*12*).

Polyamine, a small aliphatic polycationic chemical, plays a crucial role in the regulation of human skin melanogenesis (*13*). Putrescine (1,4-diaminobutane) is the smallest member of the polyamine family and is found in live mammalian cells (*14*). It is a polycationic chemical molecule produced from l-ornithine or regulated by ornithine decarboxylase (*15*). It can interact with negatively charged compounds inside the cell due to its natural substrate. Moreover, polyamines are essential for the regulation of melanogenesis in human skin. According to previous findings, putrescine can stimulate pigment production by tyrosinase in human skin explants and reduce the activity of intracellular H_2_O_2_ of polyamine catabolism (*13*). However, few studies have been conducted on the involvement of melanin synthesis activity and the mechanism of melanogenesis by putrescine. Furthermore, it is unknown whether putrescine stimulates melanin synthesis to postpone the beginning of premature greying of the hair. Therefore, the aim of this study is to investigate the impact of putrescine on the melanogenesis signalling pathway of B16F1 mouse melanoma cells.

## MATERIALS AND METHODS

### Chemicals

Dulbecco’s modified Eagle’s medium (DMEM), foetal bovine serum (FBS), trypsin–EDTA and antibiotic reagents were obtained from Gibco BRL (Life Technologies, San Francisco, CA, USA). Putrescine dihydrochloride (butane-1,4-diamine dihydrochloride), diacetyldichlorofluorescein (DCF), H_2_O_2_, vitamin C, potassium hexacyanoferrate(III), iron(III) chloride, trichloroacetic acid (TCA), tyrosinase, α-MSH, MTT (3-(4,5-dimethyl-2-yl)-2,5-diphenyltetrazolium bromide) reagent, and radio-immunoprecipitation assay (RIPA) lysis buffer were obtained from Sigma-Aldrich, Merck (St. Louis, MO, USA). The Western blot antibodies were sourced from Santa Cruz Biotechnology (Santa Cruz, CA, USA). TRIzol™ reagent was purchased from Thermo Fisher Scientific (San Jose, CA, USA). All reagents used for the experiment were of analytical grade.

### Tyrosinase activity assay

Tyrosinase activity was tested to validate the initial stage of melanin synthesis. First, 3 µL of different concentrations of putrescine (0.25, 0.5, 1 and 2 mM) were added to a mixture of 237 µL of 0.1 M sodium phosphate (pH=6.5) and 40 µL of 1.5 mM tyrosine in a microtube with continuous vortexing. Then, 20 µL of 1500 U/mL tyrosinase were added, followed by a 30-minute incubation at 37 °C. After incubation, the absorbance at *λ*=475 nm was measured with a spectrophotometer (SpectraMax M3; Molecular Devices, Sunnyvale, CA, USA). The negative control included 0.1 % vitamin C, the blank solution was Na_3_PO_4_ and tyrosine and the control contained Na_3_PO_4_, tyrosine and tyrosinase. The tyrosinase activity (%) was determined by comparing the absorbance of the putrescine group to that of the control group using the following formula:


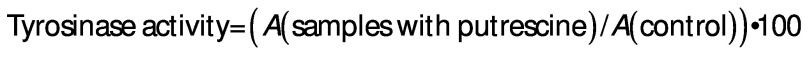
 /1/

### Cell lines and cell cultures

ATCC (American Type Culture Collection, Manassas, VA, USA) supplied a mouse melanoma cancer cell line (B16F1). The cells were cultured individually as monolayers using DMEM containing 10 % FBS with antibiotic reagents (penicillin at 10 000 U/mL and streptomycin/amphotericin at 10 000/2500 g/mL) in a 5 % CO_2_ humidified atmosphere at 37 °C.

### MTT assay

The viability of B16F1 mouse melanoma cells was assessed with putrescine (0.25, 0.5, 1 and 2 mM) using an MTT assay (*16*). B16F1 cells were seeded at a density of 10^6^ cell/mL with 10 % DMEM-FBS in 24-well plates in an incubator at 37 °C, in a 5 % CO_2_ and a humidified atmosphere. After a 24-hour treatment with different doses of putrescine, MTT reagent (5 mg/mL, 400 µL) was added and incubated for 4 h. The blank group contained the cells with DMEM supplement solution without putrescine. The insoluble purple formazan was dissolved in dimethyl sulfoxide (DMSO), and absorbance (*A*) was measured at a wavelength *λ*=570 nm using a spectrophotometer (SpectraMax M3; Molecular Devices).

### Diacetyldichlorofluorescein fluorescence assay

DCF is a widely used fluorescent biomarker for ROS production (*17*). A 96-well plate was seeded with B16F1 cells at a density of 10^6^ cell/mL. First, 250 μM of H_2_O_2_ were added to the cells to induce oxidative stress. After 24 h, DMEM containing a 20 μM 2',7'-dichlorofluorescin diacetate (DCFH-DA) solution was added and left for 20 min. Then, putrescine was added and the mixture was incubated for 1 h. The blank consisted of DMEM-FBS and DCFH-DA solutions not treated with putrescine. The control contained DMEM-FBS, DCFH-DA solution and the cells treated with 250 μM H_2_O_2_. After washing with 1× phosphate-buffered saline (PBS), 200 µL of Hank's balanced salt solution (HBSS) buffer containing 250 µM H_2_O_2_ were added. A spectrophotometer (SpectraMax M3; Molecular Devices) was used to calculate the relative H_2_O_2_ concentration at *A*_485 nm_/*A*_535 nm_ (fluorescence intensity for excitation/emission, respectively).

### Melanin production assay

Melanin was produced according to the procedure described by Kwon and Kim (*18*) with modifications. B16F1 cells (10^6^ cell/mL) were seeded in a 24-well plate with 10 % DMEM-FBS medium and incubated for 24 h at 37 °C in a 5 % CO_2_ humidified atmosphere incubator. Various concentrations of putrescine and the positive control, α-MSH, at 6 μM were added and incubated for 72 h. The blank contained DMEM supplement solution not treated with putrescine. The control contained cells treated with 250 μM H_2_O_2_ in 1× PBS. The production of melanin was examined under a microscope. Then, 400 μL of 1 M NaOH were used to dissolve the cell pellets. The cell lysates were immediately transferred to a 96-well plate at room temperature. The absorbance of the solution at *λ*=450 nm was measured using a spectrophotometer (SpectraMax M3; Molecular Devices).

### Reverse transcription polymerase chain reaction analysis

The cells at a density of 10^6^ cell/mL were seeded and incubated with the addition of H_2_O_2_. Then, they were treated with different concentrations of putrescine (0.25, 0.5, 1 and 2 mM) and incubated for 24 h. The blank consisted of DMEM supplement solution without putrescine. The control contained cells treated with 250 μM H_2_O_2_. The cells were lysed with TRIzol reagent. Total RNA was then purified using chloroform, isopropyl alcohol and 75 % ethanol. RNA concentration was measured using a UV-VIS spectrophotometer (SpectraMax M3; Molecular Devices) at *A*_260 nm_/*A*_280 nm_ wavelength. RocketScript™ reverse transcriptase (Bioneer, Daejeon, South Korea) was used with diluted RNA (0.2 µg/µL; 5 µL) to synthesize cDNA. The mRNA was amplified by PCR using T-Gradient ThermoBlock thermocycler (Biometra, Göttingen, Germany). The PCR reaction was done under the conditions of denaturation at 95 °C for 30 s, annealing at 55 °C for 30 s and synthesis at 72 °C for 60 s (30 cycles). Glyceraldehyde-3-phosphate dehydrogenase (GAPDH) was used as a reference gene. The cDNA was amplified using the following primer sequences: *Tyr*, NM_011661 (F: 5’-AACAATGTCCCAACAGG-3’, R: 5’-TGACTCTTGGAGGTAGCTGT-3’); *Mitf*, NM_0011131 98 (F: 5’-GTACTCTGATCCCCAAGTCAT-3’, R: 5’-CATCTCCAGCTCCTGTA CTC-3’); *Trp-1*, NM_001282014 (F: 5’-CAAGTGAAGCTTTCGTCTTT-3’, R: 5’-AGCAGCCATGTTGATTAG TT-3’); *Trp-2*, NM_010024 (F: 5’-ATTCCAATGACCACTGAGAG-3’, R: 5’-CCTCCACTCTTTT ACAGACG-3’) and *Gapdh*, NM_008084 (F: 5’-TCAGTGGGAAAAAGTACACC-3’, R: 5’-ATCTCCACAGCAATGTAACC-3’). The PCR products were quantified on 2 % agarose gels with Midori green (Nippon Genetics Europe, Düren, Germany). The electrophoresis bands were determined using the Davinch-Chemi™ Imaging System (Davinch-K, Seoul, South Korea) and ImageJ software v. 1.53 (*19*). The gene expression was quantified using the comparative cycle threshold method. The relative expression (RE) of the gene was calculated as follows (*20*):


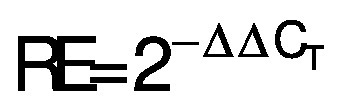
 /2/


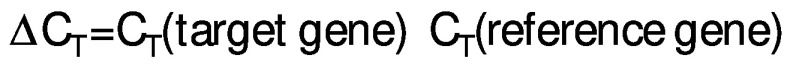
 /3/


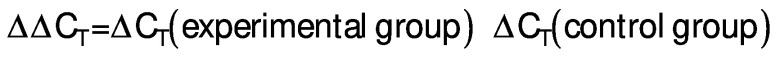
 /4/

where C_T_ is cycle threshold.

### Immunofluorescence assay

A protocol according to Jeon and Kim (*21*) was used for immunofluorescence staining. The cells (10^5^ cell/mL) were seeded in an 8-well chamber. After 24 h of incubation with H_2_O_2_, the cells were treated with putrescine (0.25, 0.5, 1 and 2 mM) and incubated with a 5 % CO_2_ atmosphere at 37 °C for 24 h. The blank consisted of DMEM supplement solution without the addition of putrescine. The control contained cells treated with 250 μM H_2_O_2_. The cells were treated with 10 % formaldehyde and fixed for 15 min. Then, they were washed with 1× PBS containing 0.5 % Tween 20, rinsed with 1× PBS containing 0.1 % Tween 20 and after that blocked for 1 h with 5 % normal donkey serum. The targeting antibodies (anti-MITF, anti-TYR, anti-TRP-1, anti-TRP-2, anti-MSRA and anti-MSRB) and secondary antibodies (donkey anti-goat conjugated FITC and donkey anti-mouse conjugated CY3) were added. The ratio of CY3 to PBS containing 0.1 % Tween 20 was 1:400, and that of FITC to PBS containing 0.1 % Tween 20 was 1:2000. The 4′,6-diamidino-2-fenilindol (DAPI) solution was added to the chamber of the slide. The iRiS Digital Cell Imaging System (Logos Biosystems, Anyang, Gyeonggi-do, South Korea) was used to determine immunofluorescence cells.

### Western blot analysis

The standard Western blotting protocols were used. The cells (10^6^ cell/mL) were then treated with putrescine at concentrations of 0.25, 0.5, 1 and 2 mM and incubated for 24 h. The blank consisted of DMEM supplement solution without putrescine. As a positive control, 6 μM α-MSH was used. The control consisted of the cells treated with 250 μM H_2_O_2_. After incubation, the cells were lysed with RIPA lysis buffer. Using a 10 % polyacrylamide gel, lysed cells were separated and placed onto a nitrocellulose membrane. After that, 5 % bovine serum albumin was added to the membrane. The anti-TYR, anti-catalase, anti-tyrosine hydroxylase, anti-MITF, anti-TRP-1, anti-TRP-2, anti-MSRA and anti-MSRB monoclonal primary antibodies (1:1000) were incubated for 2 h. The β-actin was used as a housekeeping gene for normalization. The membrane was washed and incubated with their secondary antibodies (anti-rabbit, anti-mouse and anti-goat IgG, HRP-linked antibodies; 1:5000). The WesternBright® ECL assay kit (Advansta, San Jose, CA, USA) was used to quantify the expression of proteins. The visualized protein bands were determined by Davinch-Chemi chemiluminescence imaging system (Davinch-K). ImageJ software v. 1.53 (*19*) was used to assess the protein expression levels. Relative expression (RE) of the protein was calculated by normalizing the level of the target protein band to the level of the control band. The formula used for the calculation was as follows:


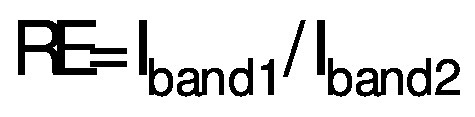
 /5/

where I_band1_=intensity of the target protein of interest band and I_band2_=intensity of the loading control band_._

### Statistical analysis

The data were presented as the mean value±standard deviation (S.D.). All experiments were performed in triplicate (*N*=3). One-way analysis of variance (ANOVA) and Duncan's multiple range test were used to examine group differences. The SPSS program v. 16 was used to conduct the statistical analysis (*22*). Statistically significant differences were indicated as follows: *p<0.05, **p<0.01 and ***p<0.001.

## RESULTS AND DISCUSSION

### Effect of putrescine on tyrosinase activity, cell cytotoxicity and intracellular H_2_O_2_ production

**Fig. 1:**
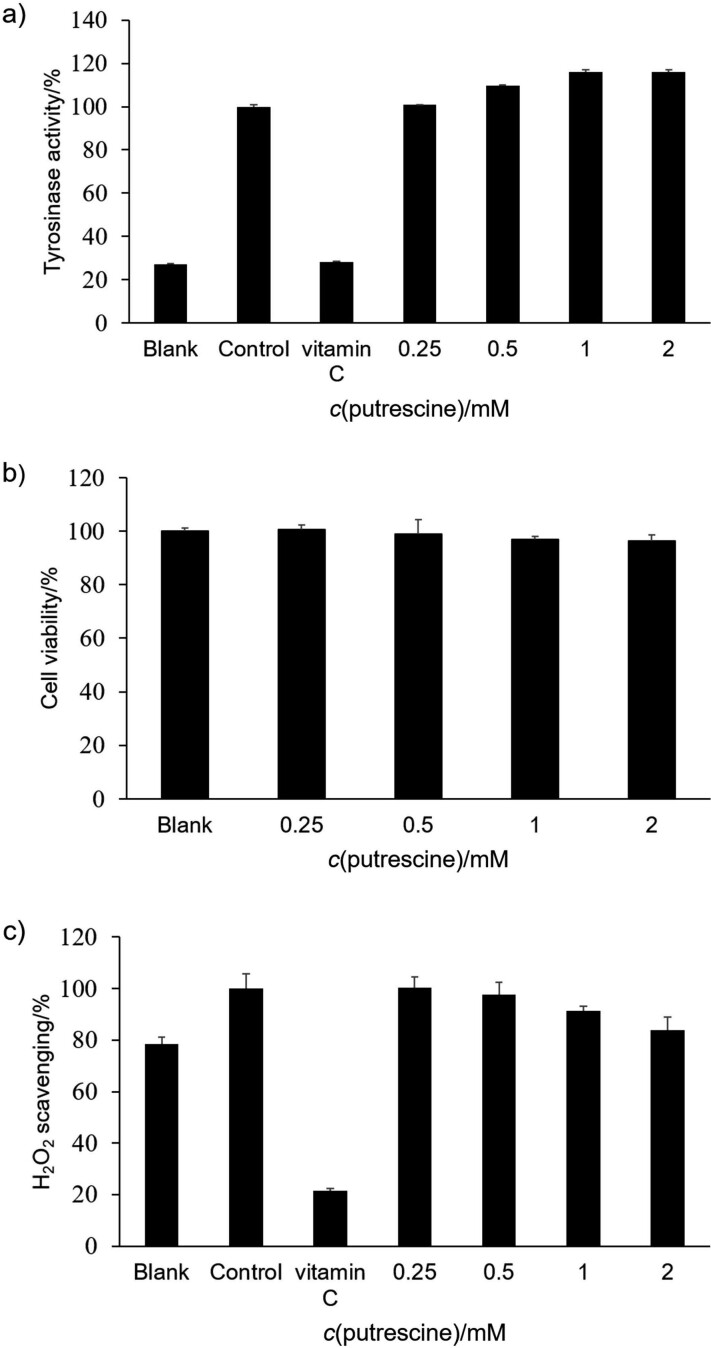
The effect of putrescine on tyrosinase activity, cell viability and H_2_O_2_ scavenging activity: a) the effect of putrescine on tyrosinase activity was examined using tyrosine as a substrate. The negative control was 0.1 % vitamin C. The blank was solution of sodium phosphate buffer and tyrosine, and the control was solution of sodium phosphate buffer, tyrosine and tyrosinase, b) the effect of putrescine on cell viability was investigated by tetrazolium dye MTT assay. The blank group was DMEM supplement solution, and c) the effect of putrescine on H_2_O_2_ scavenging activity was determined with DCFH-DA. The blank was solution of DMEM-FBS and DCFH-DA. The control was solution of DMEM-FBS, DCFH-DA and 250 μM H_2_O_2_. The control group and the sample groups differed statistically significantly: *p<0.05 and ***p<0.001 using one-way ANOVA analysis (Duncan's multiple range test)

Tyrosinase is a key enzyme in the process of melanogenesis and a critical diagnostic tool for identifying the enzyme in the melanin synthesis pathway (*23*). Commonly used to enhance skin tone and cure dermatoses, tyrosinase inhibitors may effectively suppress the formation of the pigment (*24*). The purpose of the tyrosinase activity test was to assess the function of putrescine in melanin synthesis. Fig. 1a shows a reduction in tyrosinase activity in the vitamin C-treated group that served as a negative control. Tyrosinase activity increased from 0.5 to 2 mM (p<0.001) in the group treated with putrescine, compared to the control group. Based on the direction of enzyme-oligoamine interactions, polyamines play a pivotal role in mammalian melanogenesis (*25*). In a previous study, spermidine, a polyamine compound, was reported to represent tyrosinase activity (5.1 %) at 200 μM of the spermidine-treated group (*26*). In this study, the effect of putrescine increased the activity of tyrosinase. These results suggest that putrescine may stimulate melanogenesis *via* antioxidant properties. Based on the ratio of live to dead cells, an MTT test for cell viability was performed. At these concentrations, putrescine did not cause cell death (Fig. 1b). This result indicates that putrescine is not toxic to B16F1 cells. To further investigate the potential toxicity of putrescine, putrescine concentrations of 0.25 to 2 mM were used to evaluate its effect on melanogenesis, but no evidence of cell death was observed. However, at concentrations above 10 mM, putrescine enhances cytotoxicity by considerably reducing cell viability of BT474 breast cancer cells, as shown in an earlier study (*27*). Decomposition of H_2_O_2_ can produce hydroxyl radicals, which can cause DNA damage and trigger lipid peroxidation (*28*). We used the fluorescent indicator 2',7'-dichlorodihydrofluorescein diacetate (DCFH-DA), which produces DCF and fluoresces green when it interacts with H_2_O_2_ in cells (*29*), to measure intracellular changes of H_2_O_2_ in living cells. The effect of putrescine on the scavenging activity of intracellular H_2_O_2_ was investigated in relation to the increasing H_2_O_2_ concentration during ageing and the amount of reactive oxygen species (ROS) in B16F1 cells. Fig. 1c shows that vitamin C as a negative control reduced the H_2_O_2_ by 79 %. Putrescine at the highest concentration (2 mM) reduced the concentration of H_2_O_2_ by 14 % (p<0.05). These data suggest that putrescine can inhibit the production of H_2_O_2_ by eliminating the accumulated H_2_O_2_, leading to a restoration of melanin production. In a previous study, it was reported that the concentration of spermidine was increased to 4000 μM, while the concentration of intracellular H_2_O_2_ was decreased by 83.2 % (*26*). These data suggest that putrescine can inhibit the production of H_2_O_2_ by eliminating the accumulated H_2_O_2_, leading to a restoration of melanin production. Although the use of melanoma cells provides valuable insights, especially in cancer research, it is important to note the following limitations when studying the effects of drugs on melanogenesis: melanoma cells have mutations and altered signalling pathways that are not present in normal melanocytes. These changes can significantly affect putrescine response, making it difficult to extrapolate the results to normal cells. In addition, melanoma cells often have higher proliferation rates and different growth characteristics than normal melanocytes. Therefore, the results obtained on melanoma cells may not be transferable to other types of melanocytes. Therefore, a further experiment is needed to verify the positive effect on melanin production in normal human melanocytes instead of mouse melanoma cells, which have been widely used to study melanin production but also human skin as a clinical study. Melanoma cells are cancer cells that proliferate faster than normal melanocytes. They often have genetic variations and mutations that are associated with the development of melanoma. The study of these cells could provide insights into the genetic factors that influence melanogenesis and melanoma progression.

### Impact of putrescine on melanin synthesis in B16F1 cells

**Fig. 2:**
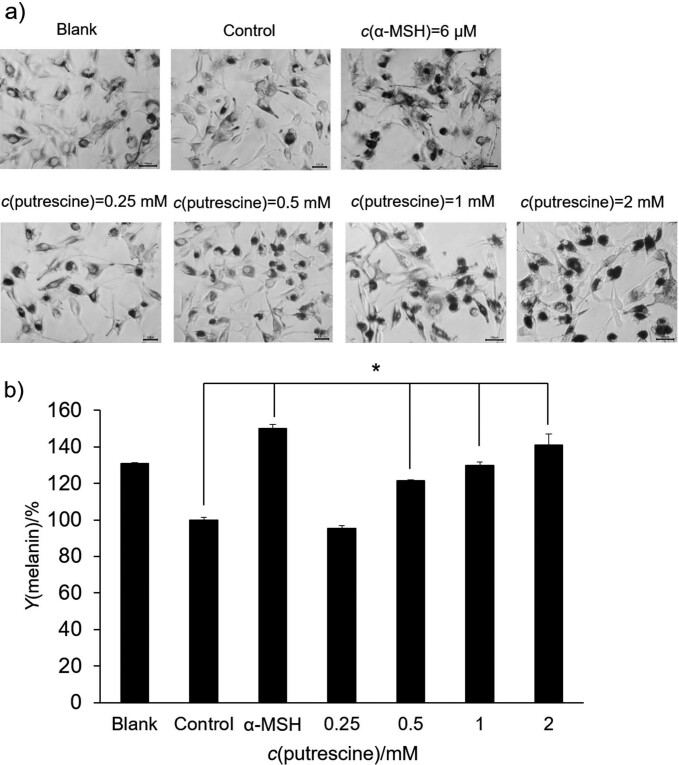
The effect of putrescine concentration on melanin production in B16F1 cells treated with 250 μM H_2_O_2_: a) the melanin production in B16F1 cells in the presence of putrescine was observed at a magnification of 200× (scale bar: 100 µm), and b) the total amount of melanin in B16F1 cells was quantified using ImageJ (*19*). The blank group was DMEM supplement solution (without H_2_O_2_). The control group contained cells treated with 250 μM sodium phosphate. Cells treated with *c*(α-MSH)=6 µM were used as a positive control. The control group and the sample groups differed statistically significantly (*p<0.05) using one-way ANOVA analysis (Duncan's multiple range test)

In general, the antioxidant activity of ageing hair follicles decreases, leading to a buildup of H_2_O_2_. An increase in H_2_O_2_ can lead to the death of melanocytes and a decrease in melanin production (*30*). As melanocytes are found in the basal cells of epidermis, skin homeostasis is highly regulated by melanogenesis, which involves the production and distribution of melanin (*1*). Throughout the ageing process, melanin plays a role in neutralising free radicals and reactive oxygen species. H_2_O_2_ accumulates in ageing hair follicles due to a decrease in antioxidants, causing melanocyte apoptosis and inhibiting melanin synthesis (*1*, *18*). To evaluate the melanogenesis of B16F1 cells in the presence of H_2_O_2_, the effect of putrescine on melanin synthesis was investigated. Fig. 2a and Fig. 2b show the production of intracellular melanin synthesis. α-MSH, a neuropeptide from the melanocortin family, is the most important activating and regulating bioactive peptide in melanogenesis. It binds to the melanocortin 1 receptor (MC1R) on the cell surface and stimulates cyclic adenosine monophosphate (cAMP), which modulates pigmentation and acts as a tyrosinase in melanocytes, leading to melanin synthesis (*31*, *32*). It is beneficial to investigate the effects of putrescine on melanin formation. Treatment with α-MSH, a positive control, increased melanin synthesis by 50 % compared to the control group. The presence of putrescine increased melanin production in a dose-dependent manner (p<0.05). In groups treated with with 0.5, 1 and 2 mM putrescine, the melanin content increased by 21, 30 and 41 %, respectively. The results of melanin synthesis show that putrescine promotes melanin formation. Similarly, putrescine increases the tyrosinase cell count (*22*). This study shows that putrescine could be used to restore melanin synthesis. Polyamine catabolism causes the activation of melanogenesis, as previous study has shown (*22*). ROS is a free radical that causes oxidative damage to lipids, DNA and proteins (*33*). Constant exposure of cell molecules to oxidative damage from free radicals generated by the body leads to a number of age-related ailments, including cancer and metabolic diseases such as hair greying (*34*, *35*). This finding suggests that putrescine massively improves the formation of melanin in cells treated with H_2_O_2_.

### Effect of putrescine on the expression of genes involved in melanin production

**Fig. 3:**
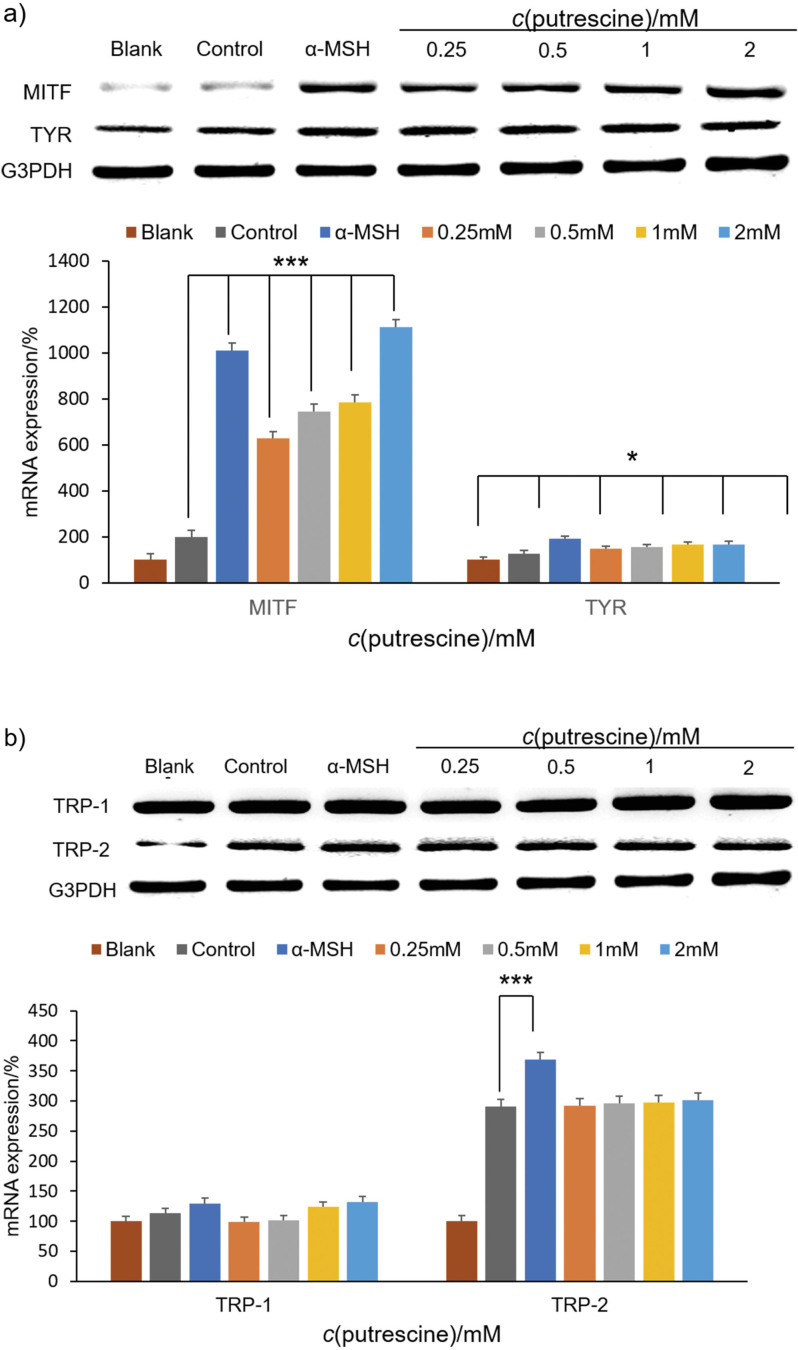
The effect of putrescine on the expression of genes involved in melanogenesis: a) *Mitf* and *Tyr*, and b) *Trp-1* and *Trp-2* in B16F1 cells treated with H_2_O_2_ were analysed in the presence of *c*(putrescine)=0.25, 0.5, 1 and 2 mM. The blank was DMEM supplement solution. The control group was treated with 250 μM H_2_O_2_. As a control for normalisation, *Gapdh* was used. The amount of mRNA was expressed as a value relative to the control group in %. The control group and the sample groups differed statistically significantly (*p<0.05 and ***p<0.001) using one-way ANOVA analysis (Duncan's multiple range test)

The effect of putrescine on gene expression (*Mitf*, *Tyr*, *Trp-1* and *Trp-2*) was determined by RT-PCR analysis. As shown in Fig. 3a and Fig. 3b, the *Mitf* and *Trp-2* genes were more highly expressed in the group treated with α-MSH than in the control group. In particular, the highest concentration of putrescine (2 mM) significantly increased the expression of the *Mitf* and *Trp-2* genes (p<0.001). Compared to the control group, the expression of the *Mitf* and *Trp-2* gene was increased by 10- and 2-fold, respectively. The expression of *Mitf* gene increased by 1012. In addition, in the group treated with putrescine the expression level of *Tyr* increased with the increase of the concentration of putrescine (p<0.05), while the *Trp-2* expression increased in the group treated with α-MSH (p<0.001). Putrescine improved melanogenesis by increasing the expression of *Mitf* and *Tyr* in B16F1 cells. These results are consistent with previous studies on polyamines and putrescine (*13*, *26*). Sridharan *et al.* (*13*) have shown that putrescine, a polyamine, stimulates human epidermal melanogenesis. The upregulation of RNA in *Tyr* and *Trp-1*, which are essential for melanogenesis, was observed in cells treated with putrescine. This suggests that putrescine promotes melanogenesis by upregulating the essential genes needed for it, thereby increasing the amount of melanin in primary melanocytes (*13*). Furthermore, Kang and Kim (*26*) reported that spermidine, a polyamine molecule, was shown to affect the expression of the *Mitf* gene. Spermidine increased expression in B16F1 cells treated with H_2_O_2_ compared to the untreated B16F1 cells, demonstrating that spermidine can regulate the process of melanin formation. These results suggest that putrescine can effectively modulate the process of melanin formation.

### Effect of putrescine on the expression of proteins invoved in melanogenesis

**Fig. 4:**
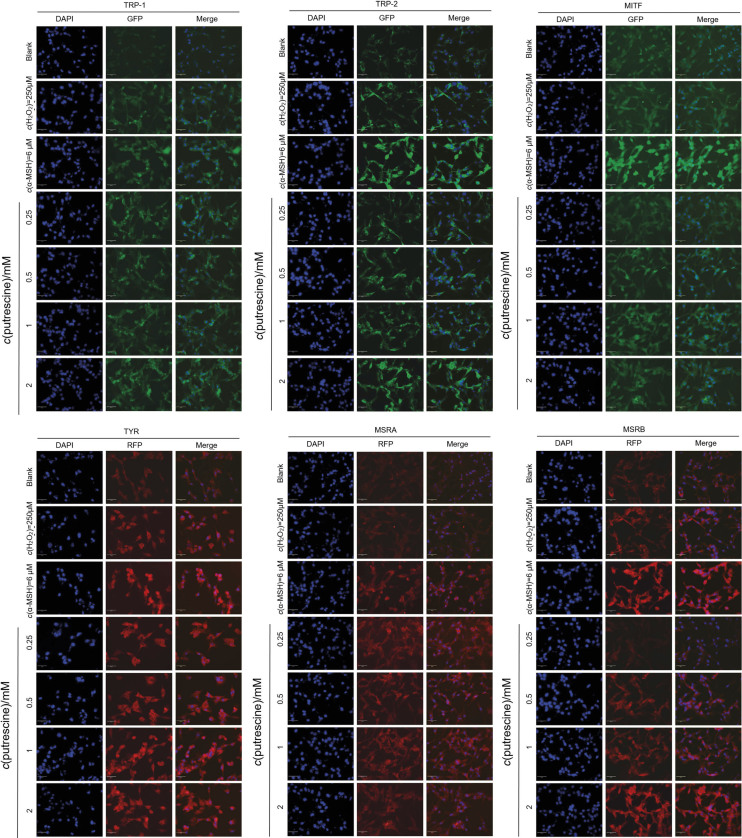
The immunofluorescence images of TRP-1, TRP-2, MITF, TYR, MSRA and MSRB proteins in B16F1 cells treated with putrescine exposed to ageing. The blank was DMEM supplement solution without putrescine. The control was 250 μM H_2_O_2_. The cells were identified using a donkey anti-goat conjugated FITC antibody labelled with a polyclonal TRP-1, TRP-2, and MITF (green signal) and donkey anti-mouse conjugated CY3 antibody with a mouse polyclonal TYR, MSRA and MSRB (red signal). The DAPI was used to stain a blue signal of cell nuclei

An immunofluorescence staining study was conducted to evaluate whether putrescine affects the expression of TRP-1, TRP-2, MITF, TYR, MSRA and MSRB proteins in B16F1 cells treated with H_2_O_2_. The fluorescence signals of DAPI, CY3 and FITC were observed as blue, red and green signal, respectively. While observing the expression of TYR, MITF, TRP-1, TRP-2, MSRA and MSRB proteins, it was concluded that the fluorescence intensity of the group treated with α-MSH, a positive control, was higher than that of the control group (Fig. 4). The expression of TYR, MITF, TRP-2 and MSRB observably increased in the group treated with 2 mM putrescine. Ko and Kim (*9*) demonstrated that H_2_O_2_ affects the decrease of melanogenesis in human melanocytes. Cells treated with H_2_O_2_ showed significantly higher expression of MSRA and MSRB than young cells, while the expression of TYR and TRP-1 was significantly lower. These results suggest that H_2_O_2_ plays a crucial role in melanogenesis by regulating antioxidant enzyme expression, and that putrescine can stimulate melanin synthesis by increasing protein expression associated with melanogenesis.

**Fig. 5:**
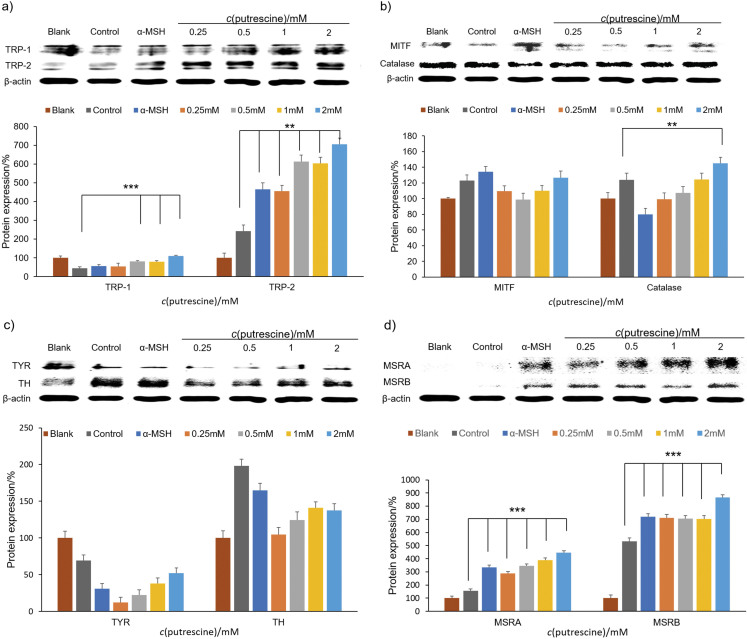
The effect of protein expression on the production of melanin by putrescine. Under H_2_O_2_-treated conditions, the TRP-1, TRP-2 (a), microphthalmia-associated transcription factor (MITF), catalase (b), TYR, TH (c), methionine sulfoxide reductases A (MSRA) and B (MSRB) (d) levels in B16F1 cells treated with *c*(putrescine)=0.25, 0.5, 1 and 2 mM were examined. The blank group was the cells containing DMEM supplement solution. As a positive control, *c*(α-MSH)=6 μM was used. The control group was the cells treated with 250 μM of H_2_O_2_. The β-actin of all groups was used to normalize the relative levels of target protein expression. The control group and the sample groups differed statistically significantly, as represented by the asterisks (**p<0.01 and ***p<0.001) using one-way ANOVA analysis (Duncan's multiple range test)

Western blot analysis was used to investigate the effect of putrescine on the expression of proteins involved in melanogenesis in B16F1 cells treated with H_2_O_2_ using antibodies against TYR, TH, MITF, TRP-1, TRP-2, MSRA, MSRB and catalase. α-MSH served as the positive control. In Fig. 5a, the groups treated with putrescine concentrations above 0.5 mM upregulated expression of TRP-1 in a dose-dependent manner (p<0.001). Moreover, putrescine significantly increased the expression of TRP-2 in a concentration-dependent manner (p<0.01). In agreement with our findings, previous studies have shown that putrescine increases the expression of TYR and TRP-1 in epidermal cells (*22*). The expression of catalase increased in the presence of 2 mM putrescine (p<0.01) (Fig. 5b). Among mentioned proteins, MITF is a key regulator of melanocyte development and skin pigmentation, since it controls the expression of TYR, the initial enzyme, TRP-1 and TRP-2 (*36*). Catalase is one of the most important antioxidant enzymes that degrade cellular H_2_O_2_ to oxygen and water, thereby significantly reducing oxidative stress (*37*). A previous study has shown that total cellular melanin content was directly associated with catalase activity in human melanocytes (*38*). Jeon *et al*. (*39*) reported that catalase in the lysosomal fraction was affected by the reduction of melanin colour by antioxidant enzymes and H_2_O_2_ treatment, suggesting a possible link between catalase and melanin production.

**Fig. 6:**
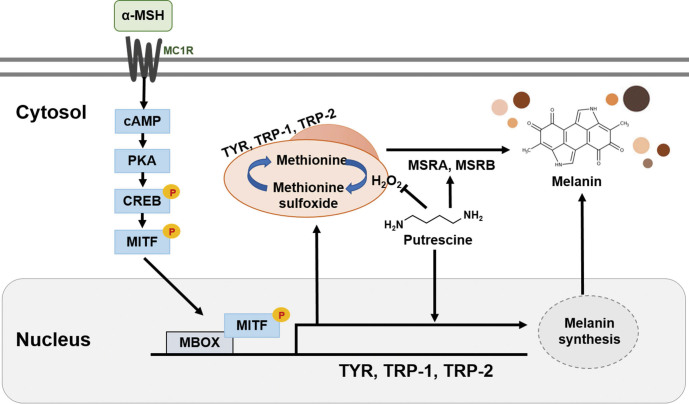
The schematic diagram of the effect of putrescine on melanogenesis stimulated by α-MSH in the MITF signaling pathway

Fig. 5c shows the expression of TYR and tyrosine hydroxylase (TH). TH is a key enzyme in the early stages of melanogenesis that catalyses the conversion of tyrosine to l-DOPA, which is a precursor for melanin synthesis (*40*). A previous study has shown that melanin content and TH activity were significantly increased in B16-F10 melanoma cells (*41*). Contrary to our expectations, the expression of TYR and TH decreased in the groups treated with putrescine compared to the control group. Nevertheless, the putrescine increased the expression levels of TYR and TH in a dose-dependent manner. Fig. 5d shows the expression of MSRA and MSRB, proteins that catalyse the enzymatic conversion of methionine sulfoxide to methionine to restore the biological function of oxidatively damaged, inactive proteins such as TYR, TRP-1 and TRP-2 (*42*). Compared to the control group treated with α-MSH, putrescine upregulated the expression of MSRA and MSRB by 234.23 and 620.56 %, respectively. In addition, MSRA expression increased in a dose-dependent manner in the presence of putrescine concentrations higher than 0.5mM (p<0.001). In particular, the group treated with putrescine at a concentration of 2 mM showed a 346 % increase in MSRA expression. Furthermore, the MSRB expression increased by 766 % in the presence of 2 mM putrescine. In a previous study, it was reported that reduced expression of glutathione reductase, catalase and MSRA, three antioxidant proteins, decreased melanin production and increased lipofuscin formation (*43*). Thus, our results suggest that the increased expression of MSRA and MSRB by putrescine may stimulate melanin synthesis. As shown in Fig. 6, the accumulation of H_2_O_2_ is a key factor that regulates the reduction of melanin synthesis in cells by inhibiting the MITF signalling pathway, which leads to the ageing of melanocytes and oxidising enzymes. However, putrescine could be used as a potential activator of expression factors such as TYR, TRP-1 and TRP-2 to modulate melanin synthesis *via* the MITF signalling pathway. It also stimulates the development of MSRA and MSRB and thus acts as a promoter of melanin production, which addresses the problem of greying hair, and offers the possibility of elucidating the ageing mechanism.

## CONCLUSIONS

Overall, it is demonstrated that putrescine, a polyamine, can induce melanogenesis by enhancing tyrosinase activity. Putrescine shows low toxicity to B16F1 mouse melanoma cells and reduces H_2_O_2_ synthesis by decreasing the expression of genes that regulates production of intracellular H_2_O_2_. The activation of protein expression factors such as TYR, TRP-1 and TRP-2 modulates melanin synthesis *via* the MITF signalling pathway and stimulates the development of MSRA and MSRB. Putrescine has the potential to be developed as a new anti-ageing ingredient and could easily be used in cosmetics designed to prevent skin ageing and premature hair greying. Our experiments certainly justify additional investment in this line of research.
